# Almond Consumption Modestly Improves Pain Ratings, Muscle Force Production, and Biochemical Markers of Muscle Damage Following Downhill Running in Mildly Overweight, Middle-Aged Adults: A Randomized, Crossover Trial

**DOI:** 10.1016/j.cdnut.2024.104432

**Published:** 2024-08-07

**Authors:** Vernon Uganiza Rayo, Maricarmen Cervantes, Mee Young Hong, Shirin Hooshmand, Nathaniel Jason, Changqi Liu, Elise North, Lauren Okamoto, Svitlana Storm, Oliver C Witard, Mark Kern

**Affiliations:** 1 School of Exercise and Nutritional Sciences, San Diego State University, San Diego, CA, United States; 2 Centre for Human and Applied Physiological Sciences, Faculty of Life Sciences & Medicine, King’s College London, London, United Kingdom

**Keywords:** human, nut, power, soreness, creatine kinase, strength, recovery

## Abstract

**Background:**

Almonds promote cardiometabolic health benefits; however, the ergogenic effect of almond supplementation on exercise recovery is less explored.

**Objectives:**

We evaluated the impacts of raw, shelled, almonds on pain, muscle force production, and biochemical indices of muscle damage and inflammation during recovery from eccentrically biased exercise.

**Methods:**

Using a randomized, crossover design, 26 healthy adults (37 ± 6 y) ran downhill (–10%) for 30 min at a heart rate corresponding to 65%–70% of maximal oxygen consumption followed by 3-d recovery periods after 8-wk adaptations to either ALMOND (2 oz/d) or isocaloric pretzel (CONTROL) feedings. Volunteers consumed the study food immediately following the run and each day during recovery. Fasted blood samples were collected, and pain and muscle function were tested before the downhill run and over 72 h of recovery.

**Results:**

Downhill running elicited moderate muscle damage (Time: *P* < 0.001; η^2^ = 0.395) with creatine kinase (CK) peaking after 24 h (CONTROL: Δ + 180% from baseline compared with ALMOND: Δ + 171% from baseline). CK was reduced after 72 h in ALMOND (Δ – 50% from peak; *P* < 0.05) but not CONTROL (Δ – 33% from peak; *P* > 0.05). Maximal torque at 120°/s of flexion was greater (Trial: *P* = 0.004; η^2^ = 0.315) in ALMOND compared with CONTROL at 24 h (Δ + 12% between trials; *P* < 0.05) and 72 h (Δ + 9% between trials; *P* < 0.05) timepoints. Pain during maximal contraction was lower (Trial: *P* < 0.026; η^2^ = 0.225) in ALMOND compared with CONTROL after 24 h (Δ – 37% between trials; *P* < 0.05) and 48 h (Δ – 33% between trials; *P* < 0.05). No differences (*P >* 0.05) in vertical jump force, C-reactive protein concentrations, myoglobin concentrations, and total antioxidant capacity were observed between trials.

**Conclusions:**

This study demonstrates that 2.0 oz/d of almonds modestly reduces pain, better maintains muscle strength, and reduces the CK response to eccentric-based exercise. This apparent effect of almond ingestion on exercise recovery has the potential to promote increased exercise adherence, which should be investigated in future studies.

This trial was registered at the clinicaltrials.gov as NCT04787718.

## Introduction

Eccentric contractions are defined as the lengthening of the muscle while under tension and have been implicated in predisposing individuals unaccustomed to exercise to muscle damage [1]. The etiology to the muscle damage is multifactorial and includes physical manipulation of skeletal muscle integrity [2] and excess oxidative stress [3]. This exercise-induced muscle damage (EIMD) is characterized by soreness, inflammation, and a temporary decrease in muscle functional capacity [1,4]. Consequently, EIMD is associated with impaired motor skill learning, which is crucial for sports performance [5] and nonadherence to exercise [6]. Thus, it is imperative to determine practical interventions to attenuate EIMD to promote physical activity adherence in the general population.

Nutritional interventions are increasingly promoted to accelerate exercise recovery and reduce risk of EIMD. Currently, multiple studies have investigated the efficacy of various dietary supplements (for example, β-hydroxy β-methylbutyrate, *ω*-3 fatty acids, collagen, etc.) on exercise recovery; however, more research is warranted given inconsistent findings [7]. Recently, a food-first approach has garnered attention likely related to cost and accessibility when compared with an exogenous supplement. For example, tart cherry juice consumption was shown to improve maximum voluntary contraction and reduce a marker of inflammation (that is, IL-6) during exercise recovery [8]. Similarly, beetroot juice consumption improved recovery of strength measurements [9] and reduced perceived muscle soreness following EIMD [10]. Thus, these data support the interest in exploring alternative functional foods to elucidate their potential role in exercise recovery.

Nuts have been gaining widespread attention due to their promising nutritive properties and benefits on cardiovascular disease, cancer, and all-cause mortality [11]. Notably, almonds consist of 19 g of protein per 100 g of nuts [12], providing both essential and nonessential amino acids including an abundant supply of branched-chain amino acids (BCAA). Regarding recovery, BCAA consumption was shown to reduce muscle soreness and maintain muscle function [13,14], as well as to reduce creatine kinase (CK) concentrations [14] following eccentric exercise. Additionally, almonds provide significant amounts of copper (Cu) and zinc (Zn), which serve structural roles in the catalytic site of superoxide dismutase (SOD), an anti-oxidative enzyme [15]. Cross-sectional analysis confirmed higher intakes of both Cu and Zn following whole almond consumption (median intake of 5g/d) when compared with nonconsumers [16]. Indeed, almond consumption (3 oz/d for 4 wk) was shown to improve antioxidant status via SOD and glutathione peroxidase expression [17]. Damage to the cell membrane of skeletal muscle, or sarcolemma, induces the suboptimal release of calcium ions that promote myocyte degradation through calcium-activated proteases [18]. This contributes to performance impairments following eccentric exercise through its effects on the length-tension relationship [2] and excitation-contraction coupling mechanisms of force production [19]. Almond consumption (∼2 oz/d for 4 wk) effectively increases plasma levels of *α*-tocopherol in adults, which is a lipid peroxide scavenger [20,21], which suggests the potential role almonds may have in maintaining membrane integrity. Thus, a scientific rationale exists regarding the potential of almonds in promoting recovery following EIMD.

In a recent study by Nieman et al. [22], significant muscle damage following exercise was reflected by a rise in CK concentrations, plasma cytokines, and muscle soreness, as well as reductions in strength and power performance. Almond consumption (∼2 oz/d for 4 wk) reduced serum CK concentrations immediately and 24-h post eccentric exercise in untrained mildly overweight, middle-aged volunteers. However, there were no benefits of either trial on muscle soreness or majority of the performance data. Perhaps a caveat of the study design was an inadequate feeding duration that was insufficient for counteracting the deleterious effects of a damaging exercise protocol in unaccustomed individuals.

Therefore, the purpose of this study is to explore the effects of almond consumption (2 oz/d) on recovery following a downhill run performed after an 8-wk feeding adaptation. We hypothesized that recovery would improve following almond consumption when compared with a control. Almonds were expected to decrease biomarkers of muscle degradation [that is, CK, myoglobin (Mgb)] and inflammation [that is, C-reactive protein (CRP)] while simultaneously improving subjective (that is, pain) and objective (that is, muscle force production) parameters of sports performance.

## Methods

### Participants

Recreationally active healthy adults (*N* = 32) were recruited from San Diego County. Those wishing to participate were required to have a body mass index between 23 and 30 kg/m^2^ and to participate in ≥1 h but not >4 h of structured exercise/wk. Individuals were excluded if they smoked, used medications (for example, nonsteroidal anti-inflammatory drugs, corticosteroids, etc.), or supplements (for example, omega-3 fatty acids, vitamin C, vitamin E, etc.) within 1 mo of participation known to affect inflammatory or antioxidant status, or had musculoskeletal limitations. These criteria were verified through a prescreening health form (Supplemental Figure 1) and the Physical Activity Readiness Questionnaire. The San Diego State University Institutional Review Board approved the protocol, and informed consent was obtained from the interested participants before initiation. Results presented in this manuscript were solely from the United States arm of a multi-centered study that included the United Kingdom [23].

### Study design

This study utilized a randomized, crossover design with two 8-wk dietary adaptations followed by a 3-d exercise recovery period, which was separated by a 4-wk washout period to prevent carryover effects. Participants were randomly assigned into either the almond trial (2 oz/d of raw almonds) or the control (isocaloric amount of unsalted pretzels) trial. Randomization was performed using a free online random number generator program by an investigator. All participants were instructed to refrain from consuming all nuts and/or seeds, as well as dietary supplements during the 8-wk feeding adaptation and exercise recovery period. Women were tested only on days 3–11 of their menstrual cycle to control for hormonal fluctuations that may impact biomarkers.

Maximal oxygen consumption (VO_2max_) was determined on a motor-driven treadmill (COSMED, or Lode) before initiating the first trial. A 3-min warm-up was provided at a self-selected speed that was subjectively rated by each participant as a moderate intensity. Following the warm-up, the incline was increased by 1% grade every minute until volitional exhaustion. Oxygen consumption, ratings of perceived exertion, and heart rate (HR) were continuously monitored during the exercise bout and recorded in 1-min increments. HR and oxygen consumption data were plotted to produce a regression line to estimate an HR reflective of 65%–70% VO_2max_ for the subsequent downhill runs. Total body fat percentage was measured via dual-energy X-ray absorptiometry before initiating the first trial for descriptive data (GE Healthcare). Daily step count was measured via actigraphy (ActiGraph GT9X) at one random timepoint during the feeding adaptation to determine physical activity levels for descriptives.

**FIGURE 1:**
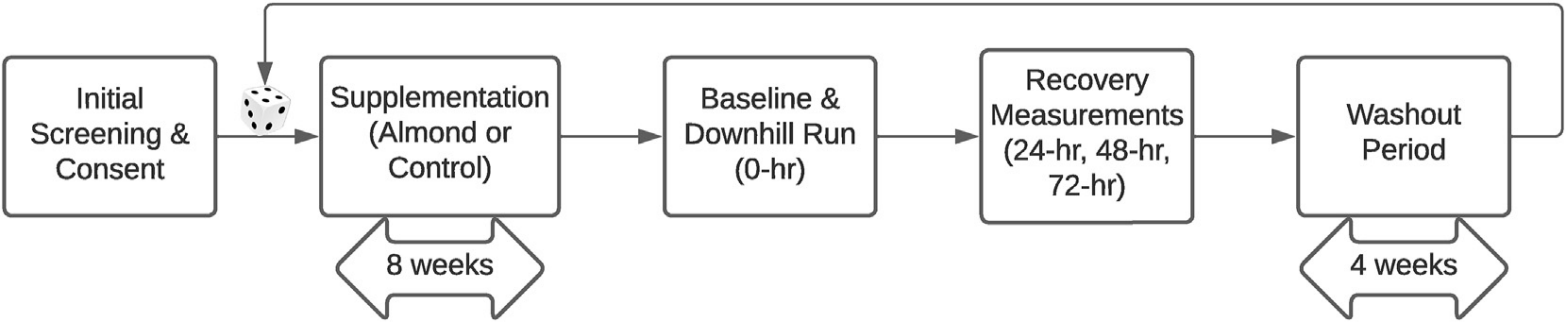
Flowchart of study design.

Following the 8-wk feeding adaptation, participants reported to the laboratory after an overnight fast and having abstained from exercise for ≥24 h. Blood samples were collected via venipuncture from the antecubital vein. The visit served as the baseline for the eccentrically biased exercise bout and 3-d recovery period (Figure 1). Participants completed a downhill run similarly as previously described [24] for 30 min at a –10% grade at an HR estimated to elicit an intensity of 65%–70% of VO_2max_ after blood collection and testing for muscle pain, strength, and vertical jump force. A serving of the test food was served immediately after the run. Participants returned to the laboratory 24, 48, and 72 h afterward to assess blood-borne and physical markers of recovery. A series of 100 mm visual analog scales (VASs) for multiple lower body muscle groups were used for subjective assessments of soreness. Quadricep and hamstring strength were determined using an isokinetic dynamometer. Vertical jump was assessed through force plate technology. Supervised feeding by an investigator continued during the following 2 d of the recovery period. After recovery testing for the first trial, subjects underwent a 4-wk washout period before beginning the other 8-wk feeding trial and subsequent recovery testing protocol.

### Study foods

Raw, shelled whole almonds (unsalted) were generously provided by the Almond Board of California (ABC). Almonds were conveniently portioned to 2-oz servings and individually packaged before shipment by the ABC. Unsalted pretzels (Snyder’s) were purchased and packaged by the investigators in a resealable, snack-sized plastic bag. A laboratory-grade scale (Ohaus Corporation) was utilized to portion the pretzels to weigh 86.5 g (∼3.0 oz). The pretzel serving provided 344 Calories, 9.4 g of protein, 1.6 g of fat, and 71.9 g of carbohydrates according to the manufacturer’s food label. The almonds provided 344 Calories, 11.5 g of protein, 30.6 g of fat, and 11.6 g of carbohydrates.

There were no specifications on when participants were allowed to consume any study foods during the 8-wk feeding adaptation. However, in terms of the recovery period, study foods were consumed under investigator supervision immediately after the downhill run, as well as 24- and 48-h after all follow-up biochemical and physical performance tests. The rationale for choosing pretzels to serve as the control was simply because it is a common, highly refined, savory snack.

### Muscle soreness

VAS measured soreness by a series of questions rating pain on both the right and left sides of the following muscle groups: quadriceps, hamstrings, gluteus, gastrocnemius, and tibialis. Additionally, pain was rated during the vertical jump and muscle force production protocols, as well as at extended (0°) and flexed (90°) knee positions while seated. Each question was followed by a 100 mm line that was demarcated at the left-most side indicating “no pain” and the right-most side indicating “worst pain imaginable.” Subjects recorded their responses by marking a spot on the line indicating their feelings about each question. Responses were quantified by measuring the distance from the left end of the line to the designated mark. Subjective ratings of pain measured via VAS have been validated in various settings, including clinical and experimental [[25], [26], [27]]. Moreover, the use of VAS for assessing perceived soreness in the nutrition and performance literature is common [8,10,13,14,[28], [29], [30]].

### Muscle force production

Maximal isokinetic strength testing of the right leg was determined using a dynamometer (Biodex Medical Systems) similarly as previously described [31]. Participants were securely strapped to the chair at the torso, waist, and ipsilateral thigh. Each trial consisted of 3 repetitions of unilateral knee flexion and extension at speeds of both 60° and 120°/s. A 1-min rest period was incorporated between the 2 different speed settings. Maximum torque [TRQ (Nm)] data for each speed was assessed before the downhill run (0 h) and during the recovery period (24, 48, and 72 h).

A force plate was used to assess the ground reaction force elicited by a maximal vertical jump protocol (Advanced Medical Technology, Inc.). Participants were instructed to be idle while standing on the force plate for 5 s. Following the countdown, participants were instructed to perform a rapid countermovement by quickly descending into a squat while swinging their arms down and back; no lead-up steps were allowed. The rapid descent was immediately followed by a maximal explosive jump; a total of 3 repetitions were allowed.

### Biochemical analyses

Fasted blood samples were collected via venipuncture before the downhill run and each morning during the recovery period. Vacutainers were centrifuged to enable the extraction of plasma from EDTA-containing tubes and serum samples. All samples were inspected for hemolysis and stored at –80°C until analysis. Plasma was assayed for CK (EKF Diagnostics), CRP (ALPCO), and Mgb (ALPCO) using commercially available ELISA kits. The serum was assayed for antioxidant capacity (Cayman Chemical) using colorimetric kits. All blood samples were batch-analyzed on the same day after all participants completed all aspects of the study.

## Statistical analyses

Data from research evaluating the effects of antioxidant supplementation on similar variables throughout recovery in 22 participants for 2 wk served as a useful model in sample size estimation with 80% power and an alpha of 0.05 [28]. All data are expressed as means ± SD. The assumption of normality was determined by the following formulas: skewness ÷ standard error of skewness, and kurtosis ÷ standard error of kurtosis. Data were considered within acceptable limits of skewness and kurtosis if the values did not exceed ± 2 [32]. Analyses were run with and without outliers to determine any changes of statistical significance. A 2 (trial) × 4 (time) within-subjects repeated-measure analysis of variance was applied to detect changes over time and between trials. Mauchley’s test of sphericity was evaluated, and violations of the assumption were adjusted with the Greenhouse–Geisser correction if the estimated epsilon was <0.75 and Huynh–Feldt correction if >0.75. Paired *t* tests were performed to further examine significant main effects as appropriate. A *P* value ≤0.05 was considered statistically significant. All data were analyzed using IBM SPSS Statistics Version 29.

## Results

### Adherence and descriptives

Of the 32 participants enrolled, 26 individuals (14 female and 12 male) completed all aspects of the study. Five individuals withdrew from our study yielding an attrition rate of 19%. The average compliance rate during the 8-wk feeding period was 90% (control) and 84% (almond). General descriptives of the population initiating the study are provided (Table 1).

**TABLE 1:** Baseline characteristics of subjects (*N* = 26) initiating the study.

Age (y)	37.2 (6.3)
Height (cm)	169.6 (9.8)
Weight (kg)	75.9 (13.0)
Body mass index (kg/m^2^)	26.3 (3.3)
Body fat (%)	33.3 (8.9)
Maximal oxygen consumption (mL/kg/min)	38.6 (12.4)
Daily step count	11,055 (5691)

Values are expressed as means (SD).

### Muscle soreness

Analysis of subjective pain ratings for the right-sided muscle groups revealed a significant time effect for the following (Table 2): quadriceps (*P* < 0.001; η^2^ = 0.492), hamstrings (*P* < 0.001; η^2^ = 0.330), gluteus (*P* < 0.001; η^2^ = 0.356), gastrocnemius (*P* < 0.001; η^2^ = 0.374), and tibialis (*P* < 0.001; η^2^ = 0.346). Additionally, a significant trial effect was observed for the right-sided quadriceps (*P* = 0.040; η^2^ = 0.164) with *post*-*hoc* tests unable to locate where the differences lie. A trend between trials was observed in the right-sided tibialis (*P* = 0.051; η^2^ = 0.150).

**TABLE 2:** Visual analog scores (mm) for pain in right-sided muscles.

Variable	0 h	24 h	48 h	72 h
Right quadriceps				
Control	12.4 (16.7)^a^	42.0 (24.2)^b^	37.5 (26.1)^b^	29.0 (23.7)^c^
Almond	7.2 (12.5)^a^	35.8 (24.7)^b^	30.8 (22.2)^b^	22.3 (19.6)^c^
Right hamstrings				
Control	11.8 (16.6)^a^	28.4 (23.3)^b^	24.4 (22.4)^b,c^	20.4 (18.9)^c^
Almond	8.1 (10.3)^a^	24.8 (24.9)^b^	22.8 (21.0)^b,c^	16.1 (13.6)^b,d^
Right gluteus				
Control	10.1 (14.6)^a^	31.8 (26.0)^b^	26.4 (22.4)^b,c^	19.6 (19.9)^c^
Almond	8.0 (11.4)^a^	29.0 (26.0)^b^	25.4 (19.6)^b,c^	18.3 (16.9)^c^
Right gastrocnemius				
Control	10.3 (14.5)^a^	25.9 (24.0)^b^	30.2 (25.4)^b,c^	23.2 (19.2)^b,d^
Almond	5.9 (8.6)^a^	25.6 (22.5)^b^	22.4 (19.5)^b^	18.7 (18.1)^b^
Right tibialis				
Control	10.1 (14.8)^a^	24.5 (22.7)^b^	28.6 (24.3)^b^	22.2 (19.0)^b^
Almond	3.6 (4.3)^a^	22.0 (23.0)^b^	21.3 (19.1)^b^	16.4 (17.3)^b^

Values are presented as means (SD). Different letters indicate significant differences (*P* ≤ 0.05) within a trial.

Pain ratings for left-sided muscle groups revealed a significant time effect for the following (Table 3): quadriceps (*P* < 0.001, η^2^ = 0.551), hamstrings (*P* < 0.001, η^2^ = 0.282), gluteus (*p* < 0.001, η^2^ = 0.406), gastrocnemius (*P* < 0.001, η^2^ = 0.400), and tibialis (*P* < 0.001; η^2^ = 0.390). Additionally, a significant trial effect was observed for the left-sided quadriceps (*P* = 0.050; η^2^ = 0.158) with post-hoc tests indicating a difference at baseline (*P* = 0.022).

**TABLE 3:** Visual analog scores (mm) for pain in left-sided muscles.

Variable	0 h	24 h	48 h	72 h
Left quadriceps				
Control	12.0 (18.2)^a,^^1^	43.6 (24.1)^b^	34.9 (24.9)^c^	29.0 (22.2)^c^
Almond	4.1 (5.6)^a,^^1^	37.3 (28.4)^b^	31.7 (25.3)^b^	22.2 (20.1)^c^
Left hamstrings				
Control	12.1 (17.2)^a^	25.3 (22.0)^b^	24.0 (21.7)^b^	21.8 (19.8)^b^
Almond	8.8 (14.1)^a^	28.6 (28.6)^b^	24.0 (21.8)^b,c^	18.3 (16.8)^c^
Left gluteus				
Control	11.0 (16.8)^a^	31.0 (26.9)^b^	28.1 (26.1)^b,c^	22.3 (21.1)^c^
Almond	4.5 (6.0)^a^	32.7 (29.9)^b^	25.3 (20.8)^b^	17.8 (17.0)^c^
Left gastrocnemius				
Control	10.3 (14.7)^a^	27.2 (24.7)^b^	31.7 (25.5)^b,c^	24.4 (19.6)^b,d^
Almond	4.4 (6.6)^a^	26.3 (25.7)^b^	24.5 (20.5)^b^	16.6 (18.7)^c^
Left tibialis				
Control	9.0 (14.2)^a^	23.3 (21.6)^b^	27.5 (24.3)^b^	20.6 (19.1)^b^
Almond	3.4 (3.7)^a^	24.9 (25.8)^b^	20.8 (18.7)^b,c^	15.2 (17.3)^c^

Values are presented as means (SD). Different letters indicate significant differences (*P* ≤ 0.05) within a trial.

1 Significant difference at respective timepoints between trials.

Pain ratings following completion of sports performance measures demonstrated a significant time effect for the following (Table 4): vertical jump (*P* < 0.001; η^2^ = 0.325), maximal contraction performed on an isokinetic dynamometer (*P* < 0.001; η^2^ = 0.439), knee flexion (*P* < 0.001; η^2^ = 0.336), and knee extension (*P* < 0.001; η^2^ = 0.441). Additionally, a significant trial effect was observed for postmaximal contraction (*P* = 0.026; η^2^ = 0.225) with post-hoc tests indicating less pain during the almond trial following 24 h (*P* = 0.012) and 48 h (*P* = 0.038) of recovery.

**TABLE 4:** Visual analog scores (mm) for pain after sports performance measures.

Variable	0 h	24 h	48 h	72 h
Vertical jump				
Control	12.3 (16.2)^a^	23.7 (23.8)^a,b^	23.0 (21.7)^b^	16.9 (17.2)^a^
Almond	6.9 (7.7)^a^	23.5 (18.9)^b^	19.5 (18.3)^b^	10.7 (10.8)^a^
Maximal contraction				
Control	12.1 (14.1)^a^	34.3 (25.1)^b,^^1^	30.6 (23.0)^b,^^1^	20.2 (20.1)^a^
Almond	7.8 (8.5)^a^	25.1 (22.5)^b,^^1^	23.0 (19.7)^b,^^1^	15.1 (15.0)^a^
Flexion				
Control	8.3 (11.8)^a^	25.4 (26.8)^b^	31.7 (27.4)^b,c^	21.9 (23.1)^b,d^
Almond	8.5 (10.3)^a^	22.7 (22.6)^b^	21.6 (20.6)^b^	13.1 (13.4)^c^
Extension				
Control	8.0 (11.8)^a^	29.2 (24.5)^b^	25.6 (22.8)^b,c^	19.1 (19.0)^c^
Almond	7.9 (10.4)^a^	21.3 (20.9)^b^	22.8 (19.3)^b^	13.3 (13.5)^c^

Values are presented as means (SD). Different letters indicate significant differences (*P* ≤ 0.05) within a trial.

1 Significant difference at respective timepoints between trials.

### Muscle force production

Significant changes in maximal TRQ were observed across various intensity settings on the isokinetic dynamometer (Table 5). A significant time effect was observed during max TRQ at 60°/s of extension (*P* < 0.001; η^2^ = 0.320) and max TRQ at 120°/s of extension (*P* < 0.001; η^2^ = 0.371). A significant trial effect (*P* = 0.004; η^2^ = 0.315) was revealed during max TRQ at 120°/s of flexion with post-hoc tests indicating higher force production following 24 h (*P* = 0.007) and 72 h of recovery (*P* = 0.006) during the almond trial. There were no significant differences observed over time or between trials during max TRQ at 60°/s of flexion. Additionally, analyses of vertical jump force revealed no significant differences over time or between trials (data not shown).

**TABLE 5:** Changes of maximum TRQ (Nm) over time across treatment groups.

Variable	0 h	24 h	48 h	72 h
Max TRQ 60° EXT				
Control	146.8 (36.7)^a^	127.0 (36.4)^b^	131.5 (36.7)^b^	138.3 (37.0)^a^
Almond	142.9 (38.2)^a^	134.9 (39.0)^b^	133.6 (38.7)^b^	138.7 (43.1)^a,b^
Max TRQ 60° FLX				
Control	77.7 (33.8)	77.1 (35.0)	81.1 (35.5)	83.4 (34.6)
Almond	78.1 (36.0)	82.5 (35.5)	81.2 (35.0)	84.0 (37.5)
Max TRQ 120° EXT				
Control	115.3 (37.5)^a^	101.6 (32.5)^b^	101.7 (39.1)^b^	106.5 (37.1)^c^
Almond	113.6 (35.5)^a^	106.4 (37.2)^b^	103.9 (37.2)^b^	105.5 (39.5)^b^
Max TRQ 120° FLX				
Control	64.1 (32.6)	63.0 (32.3)^1^	64.5 (35.7)	66.4 (33.8)^1^
Almond	68.2 (32.1)	70.3 (34.6)^1^	67.8 (35.4)	72.7 (36.0)^1^

Abbreviations: EXT, extension; FLX, flexion; TRQ, torque.

Values are presented as means (SD). Different letters indicate significant differences (*P* ≤ 0.05) within a trial.

1 Significant difference at respective timepoints between trials.

### Biochemical indices

**FIGURE 2:**
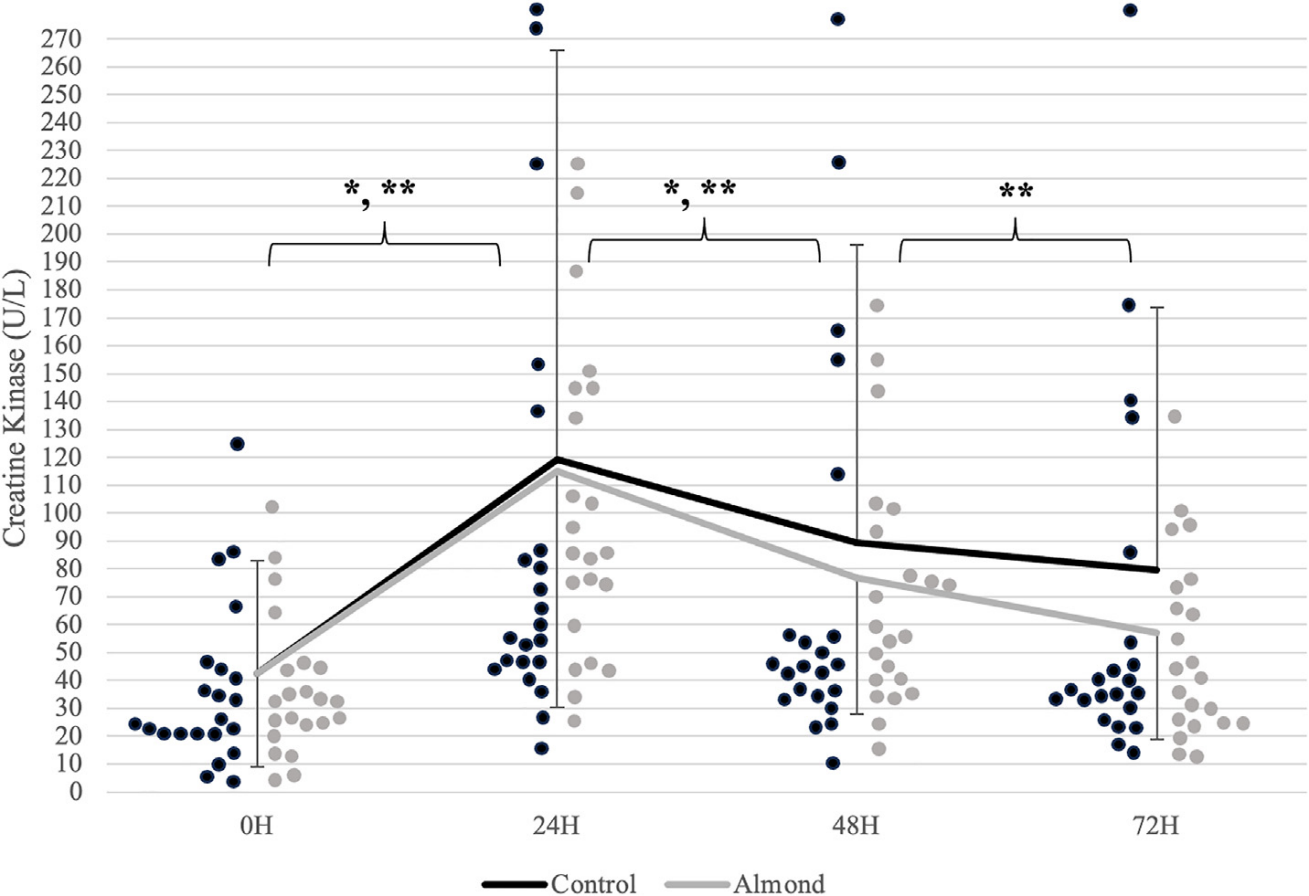
Changes in creatine kinase concentrations (units/L) over time across trials. ^1^Black (control) and gray (almond) dots represent individual data points within the respective timepoint. ∗ indicates significant difference in change between timepoints during the control trial (*P* < 0.05). ∗∗ indicates significant difference in change between timepoints during the almond trial (*P* < 0.05).

**FIGURE 3:**
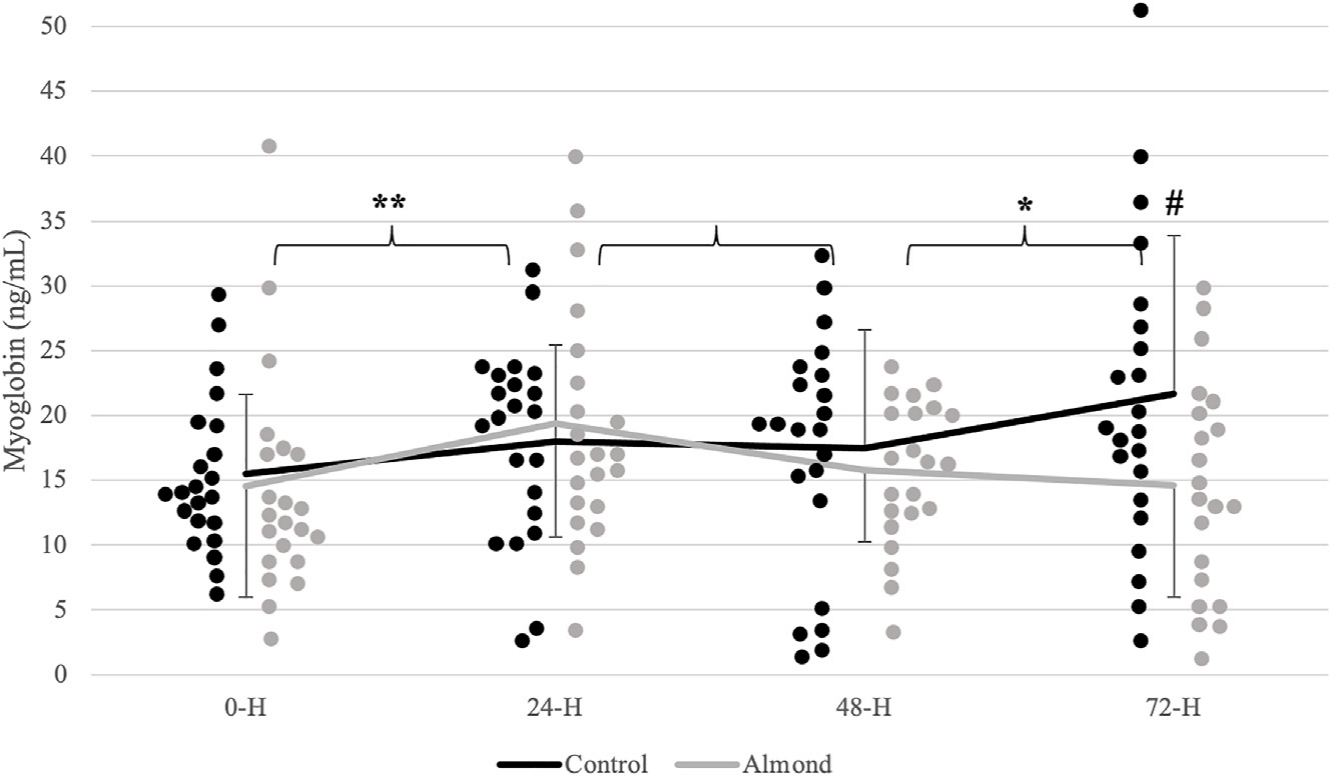
Changes in myoglobin concentrations (ng/mL) over time across trials. ^1^Black (control) and gray (almond) dots represent individual data points within the respective timepoint. ∗ indicates significant difference in change between timepoints during the control trial (*P* < 0.05). ∗∗ indicates significant difference in change between timepoints during the almond trial (*P* < 0.05). # indicates significant difference between trials at a respective timepoint (*P* < 0.05).

**FIGURE 4:**
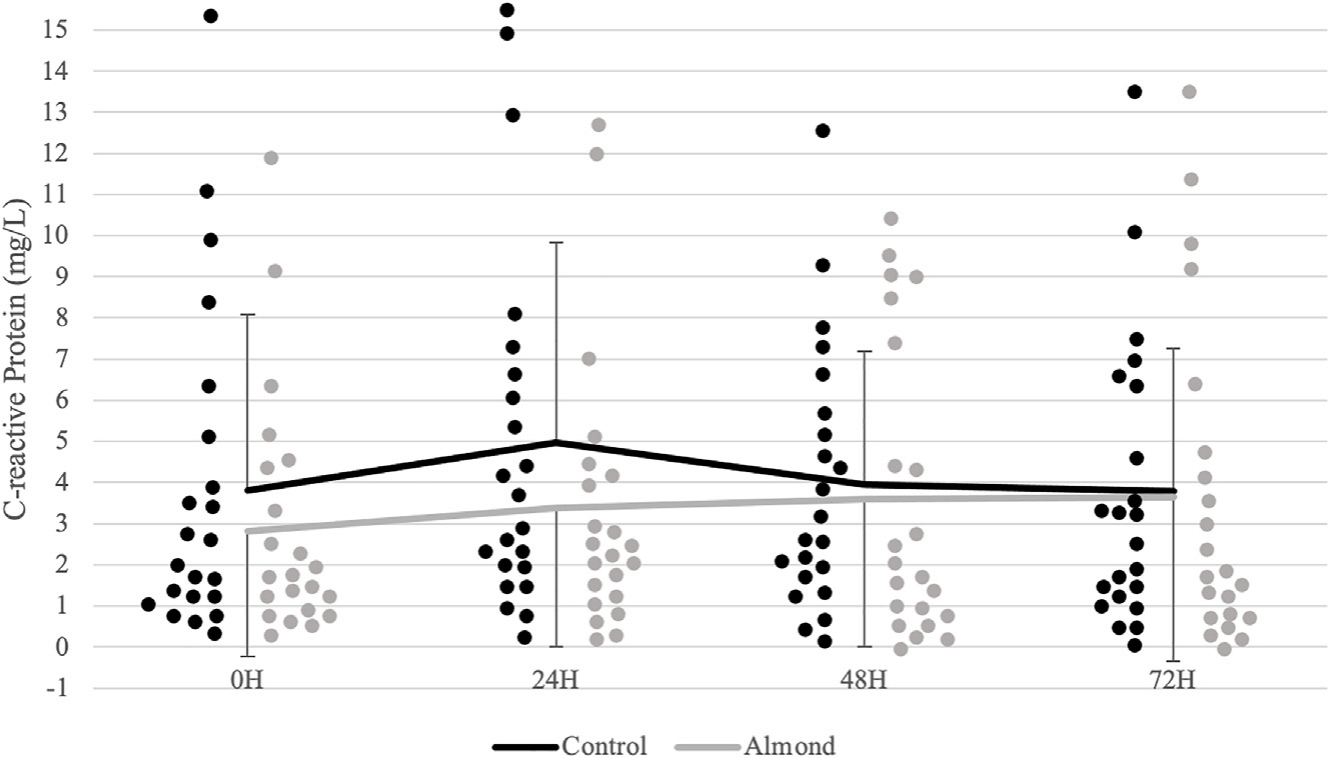
Changes in C-reactive protein concentrations (mg/L) over time across trials. ^1^Black (control) and gray (almond) dots represent individual data points within the respective timepoint.

**FIGURE 5:**
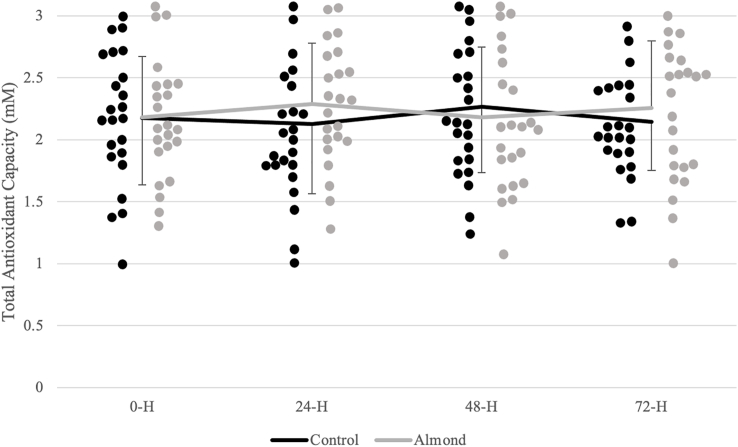
Changes in total antioxidant capacity (mM) over time across trials. ^1^Black (control) and gray (almond) dots represent individual data points within the respective timepoint.

Analyses revealed a significant time effect (*P* < 0.001; η^2^ = 0.395) for CK activity (Figure 2). CK activity was highest 24 h after the downhill run and decreased from the 24-h timepoint after 48 h of recovery in both trials. Reductions in the biomarker plateaued within the 48–72 h period during the control (*P* = 0.183), whereas a continued reduction in CK activity was observed during the almond trial (*P* < 0.001). A significant trial x time interaction (*P* = 0.036; η^2^ = 0.126) was observed for Mgb concentrations (Figure 3). Mgb did not peak until after 72 h of recovery during the control (*P* = 0.022). A peak was observed 24 h after the downhill run (*P* = 0.032) with a subsequent return to baseline after 48 h of recovery (*P* = 0.980) during the almond trial. At the 72-h timepoint, there were higher Mgb concentrations during the control relative to the almond trial (*P* = 0.006). There were no significant differences in CRP concentrations (Figure 4) and total antioxidant capacity over time and between trials (Figure 5).

## Discussion

In the present study, we compared the effects of 2-oz daily almond supplementation compared with an isocaloric pretzel feeding on postexercise recovery in healthy, mildly overweight, middle-aged adults. The downhill run elicited a moderate amount of temporary muscle damage in both study cohorts. This was evident through the transient rise in CK activity – peaking after 24 h – during the recovery period. Simultaneously, the perception of muscle soreness was highest and muscle force production was lowest 24 h following the downhill run. CK activity decreased following 48 h of recovery but plateaued thereafter during the control trial. CK reductions continued throughout the recovery period during the almond trial, but failed to return to baseline values during both trials. Relative to the control, pain during maximal contraction performed on the Biodex was less after 24–48 h of recovery in the almond trial. Additionally, Max TRQ at 120°/s of knee flexion for the almond trial was better maintained after 24 and 72 h of recovery when compared with the control. Taken together, the present study provides preliminary evidence of almond snacking serving as an ergogenic aid through modest improvements in CK clearance, pain reduction, and muscle force production.

CK is regarded as a common biochemical measurement reflective of the degree of muscle damage [33]. Thus, an increase or decrease in CK concentrations is reflective of injury or recovery, respectively. The observed pattern of a transient CK rise, increased muscle soreness, and performance impairment postexercise is consistent with a similar study examining the effects of almond supplementation (2 oz/d for 4 wk) on recovery following eccentric exercise in untrained participants [22]. Authors reported a marked increase of CK concentrations 24 h postexercise with significantly lower values in an almond-supplemented group when compared with a control [22]. Over the 4-d recovery period, CK concentrations failed to return to baseline values in either group, aligning with our results. Interestingly, our present trends in postexercise CK concentrations, soreness, and muscle function also are similar to our previous study that administered pistachio nuts as a recovery snack following a downhill run in physically active young men [29]. There were no clear benefits of pistachio supplementation on CK concentrations given that both the control and pistachio trials similarly reduced the marker of muscle damage to baseline values after 72 h of recovery following a similar downhill protocol. Thus, our present data suggest that almonds may potentiate recovery, which is reflected by a modest reduction in CK concentrations. However, it appears ineffective at lowering the marker of muscle damage to baseline values in a population of untrained individuals following eccentric exercise.

In this study, inflammatory status was unaffected, as evidenced by nonsignificant changes in CRP concentrations and total antioxidant capacity during recovery. When compared with another functional food, ingesting grape juice 2 h pre-exercise increased total antioxidant capacity but did not alter CRP concentrations following a run to exhaustion in recreational male runners [34]. Similarly, consumption of Montmorency cherry juice did not reduce CRP concentrations over a 72-h recovery period following prolonged, intermittent sprint activity in male soccer players. [8]. Effects on CRP following pistachio supplementation are mixed, showing no changes [30] or a minor benefit in recreationally active males [29]. This observation poses the question of different nutrient characteristics and phytochemical profiles that promote varying degrees of alterations to oxidative status. Altogether, this suggests different anti-inflammatory mechanisms that contribute to the protective effects of almonds that were unexplored in our study.

Peak pain scores ranged from 20 to 40 on a 0–100 VAS, suggesting that pain elicited by muscle damage was relatively minor. There was no apparent benefit from almond supplementation on pain experienced in both left- and right-sided muscle groups. Pain ratings did not return to baseline values in any muscle group following 72 h of recovery during both trials. However, pain during maximal contraction performed on the Biodex was less after 24–48 h of recovery in the almond trial when compared with the control. Additionally, muscle force production at 120°/s of knee flexion for the almond trial was better maintained after 24 and 72 h of recovery when compared with the control. The observations of the present study suggest a modest benefit of almond consumption on pain reduction and maintenance of muscle function. Comparatively, pistachio supplementation (3 oz/d for 4 wk) reduced pain ratings experienced on both left- and right-sided quadriceps, hamstrings, and gluteus following a downhill run in recreationally active males [29]. Additionally, max TRQ at 120°/se of knee flexion was better maintained throughout recovery during the pistachio trial, aligning with our findings. Variability in pain ratings between the present study and the noted pistachio study may be related to the differing study population composed of physically active males who may have developed exercise training adaptations.

Mgb is considered a short-term marker of muscle damage in the blood as it is reported to peak 1–3 h after exercise [35]. Therefore, interpreting the results after a longer period is challenging. In our study, Mgb peaked 24 h after the downhill run and returned to baseline values after 48 h during the almond trial, somewhat aligning to its acute nature. Unexpectedly, Mgb concentrations remained constant 24–48 h postexercise and peaked after 72 h of recovery during the control trial. Nieman et al. [22] observed a marked increase in Mgb concentrations ∼5 min following eccentric exercise that remained elevated throughout a 4-d recovery period in both a control and almond group. These findings suggest that there is no clear benefit of almond supplementation on Mgb concentrations during recovery from exercise. A possible explanation of our Mgb trend during the control trial is that the carbohydrate content of the pretzels (∼72 g per study serving) may have delayed the Mgb response. Mgb concentration decreased immediately after and 1-h post intense exercise (60 min at 85% VO_2max_) following consumption of a carbohydrate (75 g) beverage in well-trained male runners [36]. Authors cited a possible explanation of the reduced putative marker of muscle damage following carbohydrate supplementation may have been attributed to attenuation in muscle damage itself that is evident through a blunted inflammatory response. Carbohydrate supplementation was shown to decrease the production of cytokines involved in the secondary inflammatory cascade following exercise, IL-10 and IL-1 receptor agonist (IL-1ra) [37,38]. Additionally, postexercise total leukocyte count was reduced following carbohydrate supplementation [38], indicating a decreased immune response to repair damaged tissues. Authors also postulated that carbohydrate ingestion promotes Mgb clearance from circulation possibly through hormonal alterations [36]. Consumption of a glucose solution immediately before moderate-intensity exercise reduced plasma epinephrine concentrations with authors suggesting an inhibitory effect of carbohydrate ingestion on plasma catecholamines [39,40]. Peak et al. [36] concluded that a reduced epinephrine concentration maintains renal blood flow during exercise; consequently, promoting clearance of Mgb from the bloodstream. Given its acute nature, our Mgb data requires scrutiny and future research should consider more frequent measurements of this biomarker in the immediate postexercise period. Additionally, indicators of exercise immunology such as cytokines (for example, IL-10 and IL-1ra), hormones (for example, epinephrine), and leukocytes should be considered for a deeper understanding of the extent of muscle damage and postexercise recovery.

Strength of our study was the incorporation of an accessible simple food as a snack into a habitual diet, rather than an isolated supplement. Additionally, the implementation of a downhill run is an ecologically valid mode of eccentric-based exercise. Our muscle damage protocol may raise questions given the crossover design that possibly promoted a repeated bout effect [41]. In theory, participants may adapt to a single bout of eccentric exercise, consequently gaining some protection from subsequent bouts. Studies have suggested the beneficial effects continue for ≤5–6 wk [42,43]. Our downhill runs were separated by ≥12 wk (4-wk washout plus 8-wk food adaptation). Thus, we are confident that a repeated bout effect unlikely impacted our findings. However, given the unfamiliarity of eccentric muscle damage in a population of untrained individuals, our 72-h recovery may have been of inadequate duration. Garnier et al. [44] examined the differences in pain perception between downhill (–15%) and level running for 45 min without the inclusion of an ergogenic intervention in recreationally trained participants. Soreness in the quadriceps and gluteus took ≤96 h to be alleviated and returned to baseline values. Despite differences in our protocols, patterns of recovery following downhill running in an unaccustomed population may align similarly to a higher intensity protocol in recreationally trained individuals. Although almonds modestly reduced pain during sports performance measures in our study, ratings failed to return to baseline levels when analyzing right- and left-sided muscle groups. Thus, future studies should consider a recovery period that enables the observation of pain trends past 72 h to determine if almonds shorten absolute recovery time in comparison to pretzels or similar control feedings.

In conclusion, based on our findings, almond supplementation modestly improves subjective pain ratings during maximal contraction, reduces CK concentrations, and better maintains muscle force production following eccentric exercise. Taken together, our findings provide preliminary evidence to support the notion that almonds may serve as an effective functional food to facilitate recovery from muscle-damaging exercise.

## Acknowledgments

We thank the contributions of Ana Beatriz Martin and Michelle Tsang, who assisted in conducting and evaluating this research.

### Author contributions

The authors’ responsibilities were as follows – OCW, MK: designed research; VUR, MC, MYH, SH, NJ, CL, EN, LO, SS, OCW, MK: conducted research; VUR, SH, MK: analyzed data and performed statistical analysis; VUR, SH, MK: wrote the article; VUR, SH, OCW, MK: had primary responsibility for final content; and all authors: read and approved the final manuscript.

### Conflict of interest

The authors report no conflicts of interest.

### Funding

This study was supported by the Almond Board of California (Principal Investigator: MK; Co-Principal Investigator: CL, MYH, OCW, and SH). The funding source did not have any involvement in the conduct of the research and preparation of the article.

### Data sharing

Data described in the manuscript, code book, and analytic code will be made available upon request pending approval.
