# Supplementary figures and images for: Almond Consumption Modestly Improves Pain Ratings, Muscle Force Production, and Biochemical Markers of Muscle Damage Following Downhill Running in Mildly Overweight, Middle-Aged Adults: A Randomized, Crossover Trial
Source: Curr Dev Nutr. 2024 Aug 7;8(9):104432. doi: 10.1016/j.cdnut.2024.104432 (PMC11381864; doi:10.1016/j.cdnut.2024.104432)

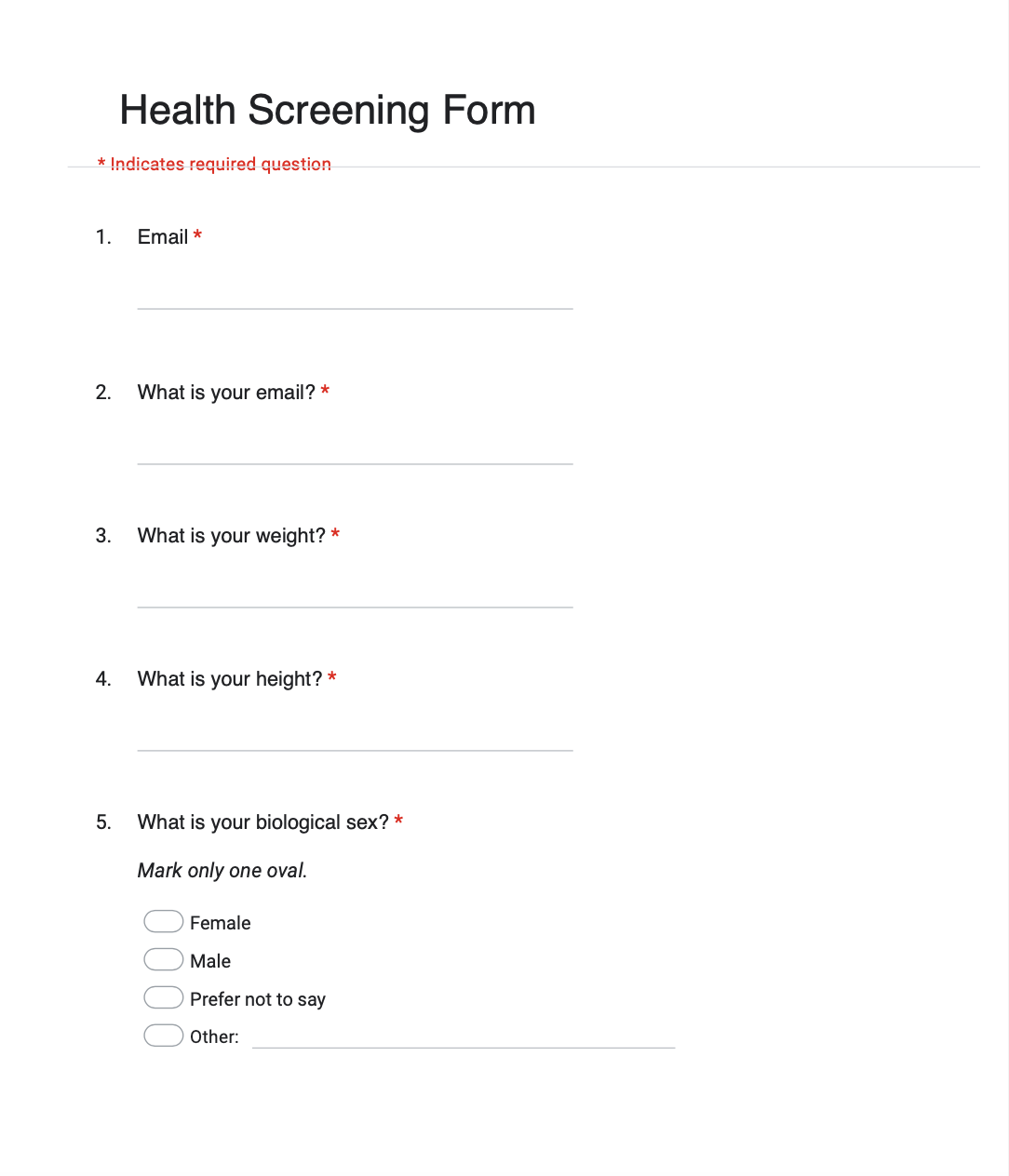


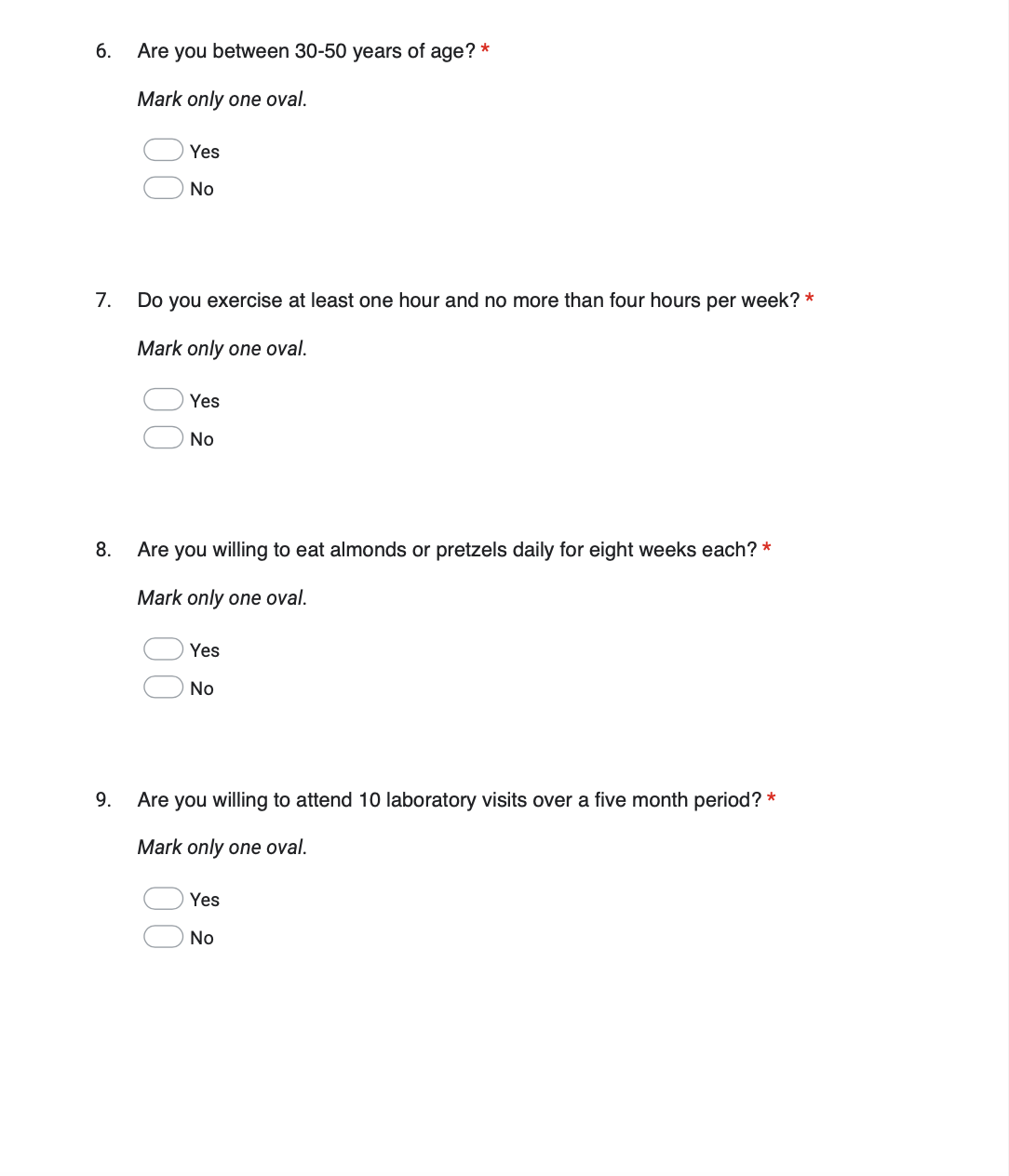


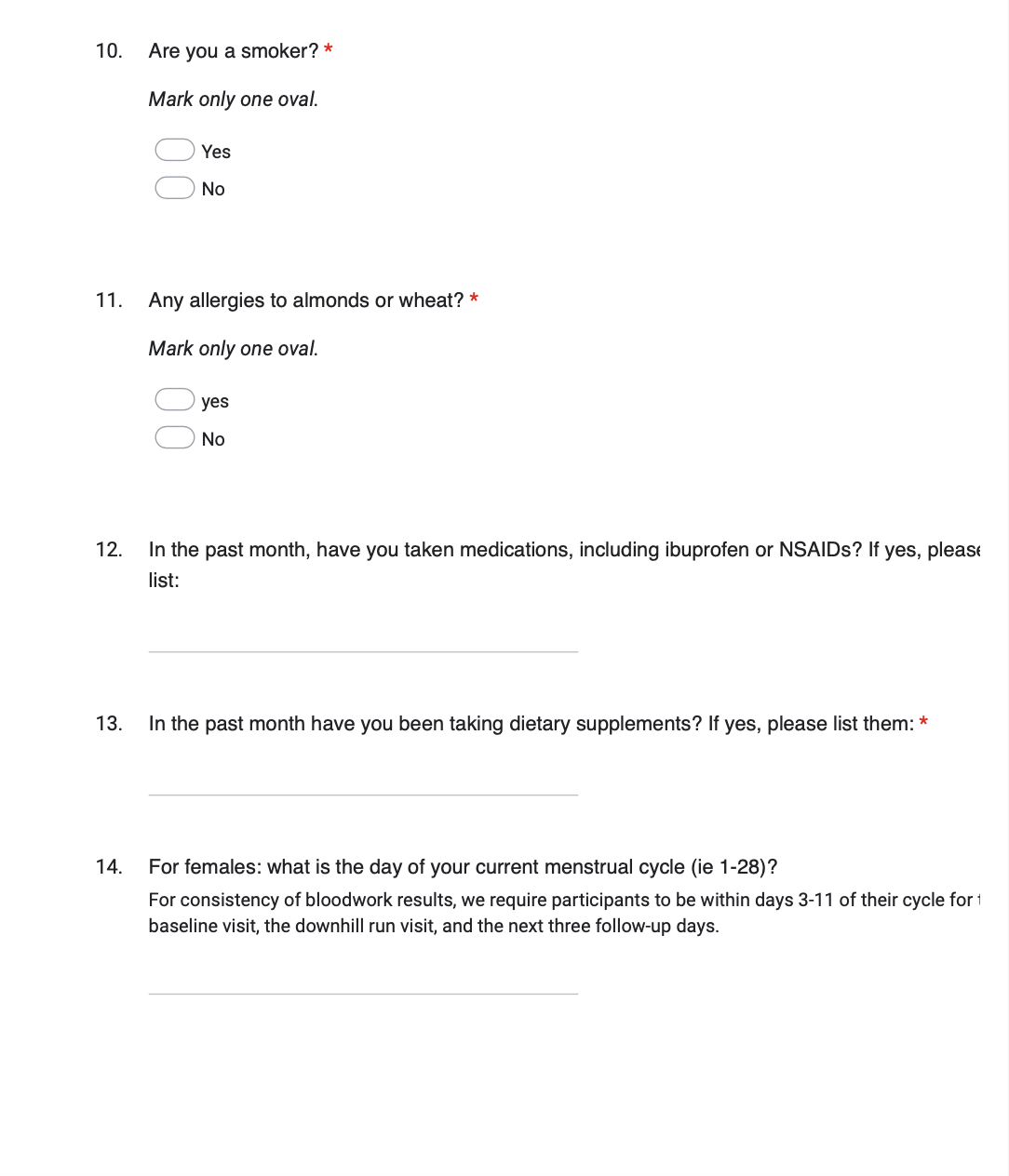


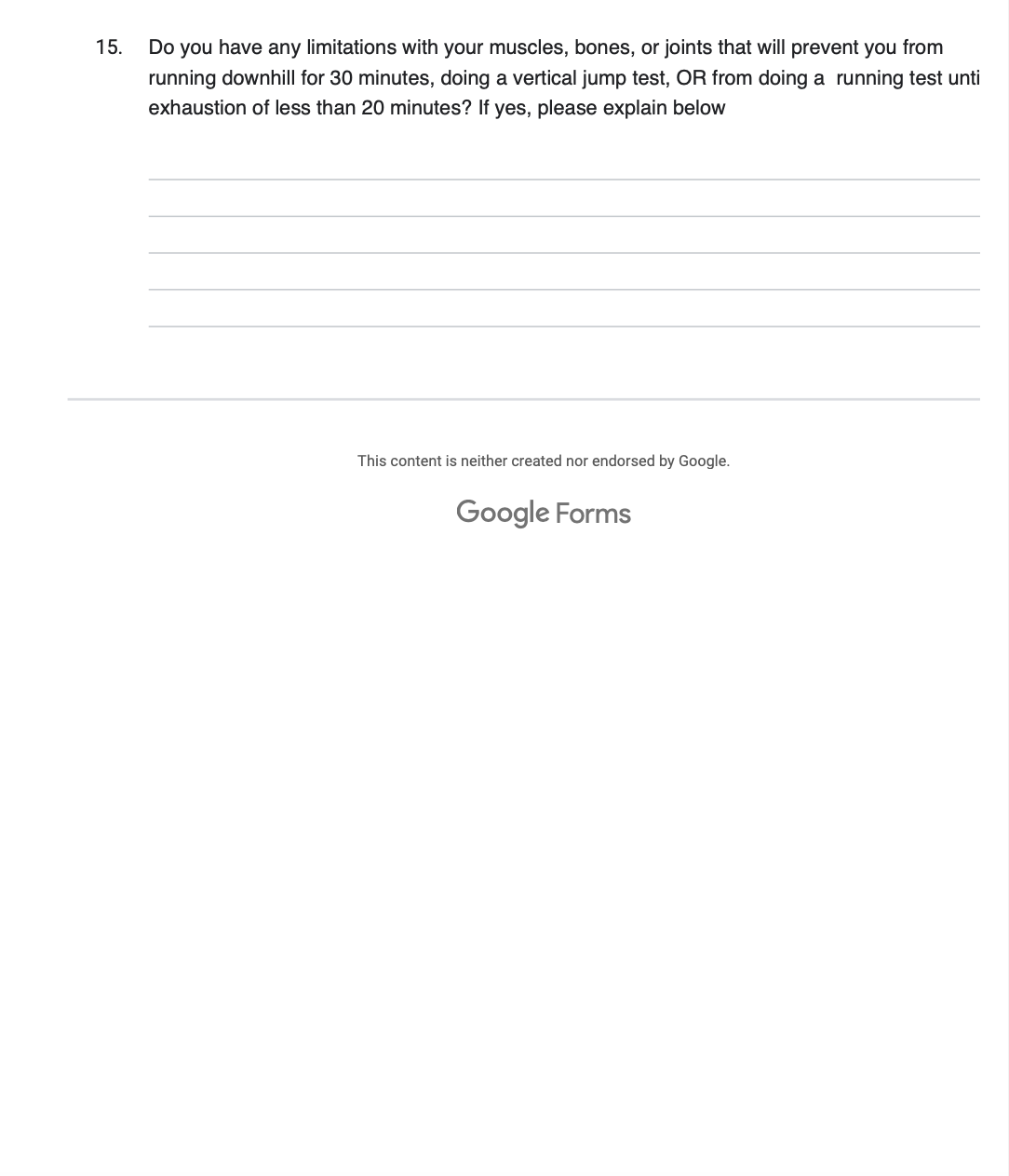

Supplement: Multimedia component 1 [file mmc1.docx]
